# Human Umbilical Cord Mesenchymal Stem Cells Extricate Bupivacaine-Impaired Skeletal Muscle Function via Mitigating Neutrophil-Mediated Acute Inflammation and Protecting against Fibrosis

**DOI:** 10.3390/ijms20174312

**Published:** 2019-09-03

**Authors:** Wen-Hong Su, Ching-Jen Wang, Hung-Chun Fu, Chien-Ming Sheng, Ching-Chin Tsai, Jai-Hong Cheng, Pei-Chin Chuang

**Affiliations:** 1 Department of Medical Research, Kaohsiung Chang Gung Memorial Hospital, Kaohsiung 833, Taiwan (W.-H.S.) (C.-C.T.); 2 Stem Cell Research Core Laboratory, Department of Medical Research, Kaohsiung Chang Gung Memorial Hospital, Kaohsiung 833, Taiwan; 3 Department of Orthopedics, Kaohsiung Chang Gung Memorial Hospital and Chang Gung University College of Medicine, Kaohsiung 833, Taiwan; 4 Center for Shockwave Medicine and Tissue Engineering, Kaohsiung Chang Gung Memorial Hospital, Kaohsiung 833, Taiwan; 5 Department of Obstetrics and Gynecology, Kaohsiung Chang Gung Memorial Hospital and Chang Gung University College of Medicine, Kaohsiung 833, Taiwan; 6 Department of Pediatrics, Kaohsiung Chang Gung Memorial Hospital, Kaohsiung 833, Taiwan; 7 Department of Biotechnology, Kaohsiung Medical University, Kaohsiung 807, Taiwan

**Keywords:** umbilical cord mesenchymal stem cells, neutrophils, inflammation, fibrosis, skeletal muscle injury

## Abstract

Skeletal muscle injury presents a challenging traumatological dilemma, and current therapeutic options remain mediocre. This study was designed to delineate if engraftment of mesenchymal stem cells derived from umbilical cord Wharton’s jelly (uMSCs) could aid in skeletal muscle healing and persuasive molecular mechanisms. We established a skeletal muscle injury model by injection of myotoxin bupivacaine (BPVC) into quadriceps muscles of C57BL/6 mice. Post BPVC injection, neutrophils, the first host defensive line, rapidly invaded injured muscle and induced acute inflammation. Engrafted uMSCs effectively abolished neutrophil infiltration and activation, and diminished neutrophil chemotaxis, including Complement component 5a (C5a), Keratinocyte chemoattractant (KC), Macrophage inflammatory protein (MIP)-2, LPS-induced CXC chemokine (LIX), Fractalkine, Leukotriene B4 (LTB4), and Interferon-γ, as determined using a Quantibody Mouse Cytokine Array assay. Subsequently, uMSCs noticeably prevented BPVC-accelerated collagen deposition and fibrosis, measured by Masson’s trichrome staining. Remarkably, uMSCs attenuated BPVC-induced Transforming growth factor (TGF)-β1 expression, a master regulator of fibrosis. Engrafted uMSCs attenuated TGF-β1 transmitting through interrupting the canonical Sma- And Mad-Related Protein (Smad)2/3 dependent pathway and noncanonical Smad-independent Transforming growth factor beta-activated kinase (TAK)-1/p38 mitogen-activated protein kinases signaling. The uMSCs abrogated TGF-β1-induced fibrosis by reducing extracellular matrix components including *fibronectin-1*, *collagen (COL) 1A1*, and *COL10A1*. Most importantly, uMSCs modestly extricated BPVC-impaired gait functions, determined using CatWalk™ XT gait analysis. This work provides several innovative insights into and molecular bases for employing uMSCs to execute therapeutic potential through the elimination of neutrophil-mediated acute inflammation toward protecting against fibrosis, thereby rescuing functional impairments post injury.

## 1. Introduction

Musculoskeletal disorders and diseases are the leading causes of physical disabilities [1]. A traumatic muscle injury is a common type of musculoskeletal disorder and can greatly impair an individual’s ability to function and participate in occupational activities and those of daily life. Skeletal muscle has a good regenerative capacity; however, extensive muscle injury, such as that instigated by trauma associated with a loss of healthy muscular tissues or development of fibrous scar tissue, might prevent complete regeneration, particularly in terms of functional recovery [2]. Thus, this highlights the need to comprehensively understand how pathologic muscle processes occur, with a focus on delineating therapeutic interventions to facilitate muscle healing along with improving the extent of functional recovery post injury.

Often, an injured muscle heals slowly, and numerous growth factors, cytokines/chemokines, and signaling molecules participate in the stages of healing. The healing process can be divided into three distinct but overlapping phases: muscle degeneration and inflammation, muscle regeneration, and fibrosis [3]. Briefly, when a muscle injury occurs, it is characterized by the rupture and ensuing necrosis of the myofibers. The necrosis is triggered by disruption of myofiber sarcolemma, resulting in increased myofiber permeability and muscle degeneration, consequently activating the inflammation. Neutrophils are the first immune cells to arrive at the injured site and initiate acute inflammation, followed by sequential increases in macrophages [4]. Activated resident neutrophils can further release a variety of factors such as pro-inflammatory cytokines including tumor necrosis factor (TNF)-*α*, interleukin (IL)-1β, IL-1*α*, monocyte chemoattractant proteins, leukotrienes, or chemokines, including C-X-C motif chemokines [4,5,6]. The peripheral neutrophils can be further recruited by chemotaxis at the injury site to create a positive feedback loop and amplify the inflammation. Subsequently, myogenic satellite cells residing in the necrotic areas divide, differentiate, and fuse with pre-existing muscle fibers to repair the damaged muscle and to enhance muscle-fiber hypertrophy [7]. Furthermore, the generation of fibrotic scar tissue, accompanied by the progressive loss of muscle function, involves myostatin, interferon (IFN)-γ, and transforming growth factor-beta (TGF-β), which are released from the inflammatory cells and stimulate the production of extracellular matrix components [8]. While fibrosis initially supports the injured muscle, the sustained expansion of the collagen deposition restricts the regenerative potential of the muscle [8]. Strategies that were developed to repair damaged muscles include physical therapies [9]. and the development of molecular signaling-based strategies that can block individual trophic factors [10,11]. However, these strategies either did not elicit the desired response or failed to completely restore the impaired function in response to muscle injury.

Therapeutic advances of mesenchymal stem cells (MSCs) were attempted to treat chronic and age-related diseases, neurodegenerative disorders, wound healing, and myocardial infarction [12,13,14,15,16]. These cells are found in many tissues, including bone marrow, adipose, umbilical cord blood, and adult organs. MSCs are known as an undifferentiated population, capable of self-renewal with sustained proliferation in vitro; they are able to differentiate into multiple lineages. We previously demonstrated that MSCs are present in human umbilical cord blood (HUCB) and that they can differentiate into bone nodules in vitro, as well as into bony callus when transplanted in the femoral segment of a mice model [17]. However, these MSCs represent < 1% of all HUCB cells; thus, it is difficult to collect enough for use in clinical applications. Fortunately, a larger population of MSCs was found in umbilical cord Wharton’s jelly (WJ uMSCs) [18,19]. These uMSCs have a shorter population doubling time and can be easily cultivated in vitro. They are also well tolerated by the immune system, thus facilitating the engraftment of uMSCs into non-immune-suppressed animals without acute immune rejection [18]. Most importantly, uMSCs are easily retrieved, which alleviates the need for invasive bone marrow biopsies, with no ethical issues. Given these advantages, uMSCs are a desirable material in tissue-engineering and cell-based therapies. Of note, studies demonstrated that MSCs have the potential to differentiate into muscle-like cells. Nunes et al. showed that HUCB MSCs can acquire a muscle-like phenotype [20]. Conconi et al. also demonstrated that stem cells derived from WJ could in vitro differentiate into myoblast-like cells and in vivo differentiate into skeletal muscle cells [21]. However, previous studies on the recovery process of muscle injury by MSCs focused on myocyte differentiation or muscle regeneration; the aforementioned studies did not illustrate whether muscular function could be rescued after MSC transplantation or what healing mechanisms might be involved post engraftment of MSCs.

Of interest, although acute inflammation is considered as the body’s defensive response to muscle injury, it was also suggested that the inflammatory reaction may exacerbate muscular damage and subsequently increase the possibility of scarring or fibrosis, which is the leading cause of impairment of muscle function [22]. Neutrophils are the first line of the immune defensive system, penetrating the body’s physical barriers, and they are the first subpopulation of immune cells to appear at the injury site. Lately, accumulated evidence showed that neutrophils display a crucial role in initiation of the acute inflammatory response and promote muscle fiber damage post injury [4,23,24]. In that regard, it is hypothesized that limiting certain aspects of early-onset inflammation, led by inflammatory cells, may mitigate muscle damage and prevent subsequent muscular fibrosis. To address this issue, herein, we established a skeletal muscle injury model via injection of a local anesthetic, bupivacaine hydrochloride (BPVC), into the quadriceps muscles of C57BL/6 mice [25,26]. We report a study designed to elucidate if the transplantation of MSCs derived from umbilical cord Wharton’s jelly (uMSCs) has regulatory properties on the skeletal muscle healing process, especially through restriction of neutrophil-derived acute inflammation toward protecting against fibrosis, thus restoring functional impairment post muscle injury.

## 2. Result

### 2.1. Characterization of Mesenchymal Stem Cells Derived from Wharton’s Jelly of Human Umbilical Cord (uMSCs)

**Figure 1:**
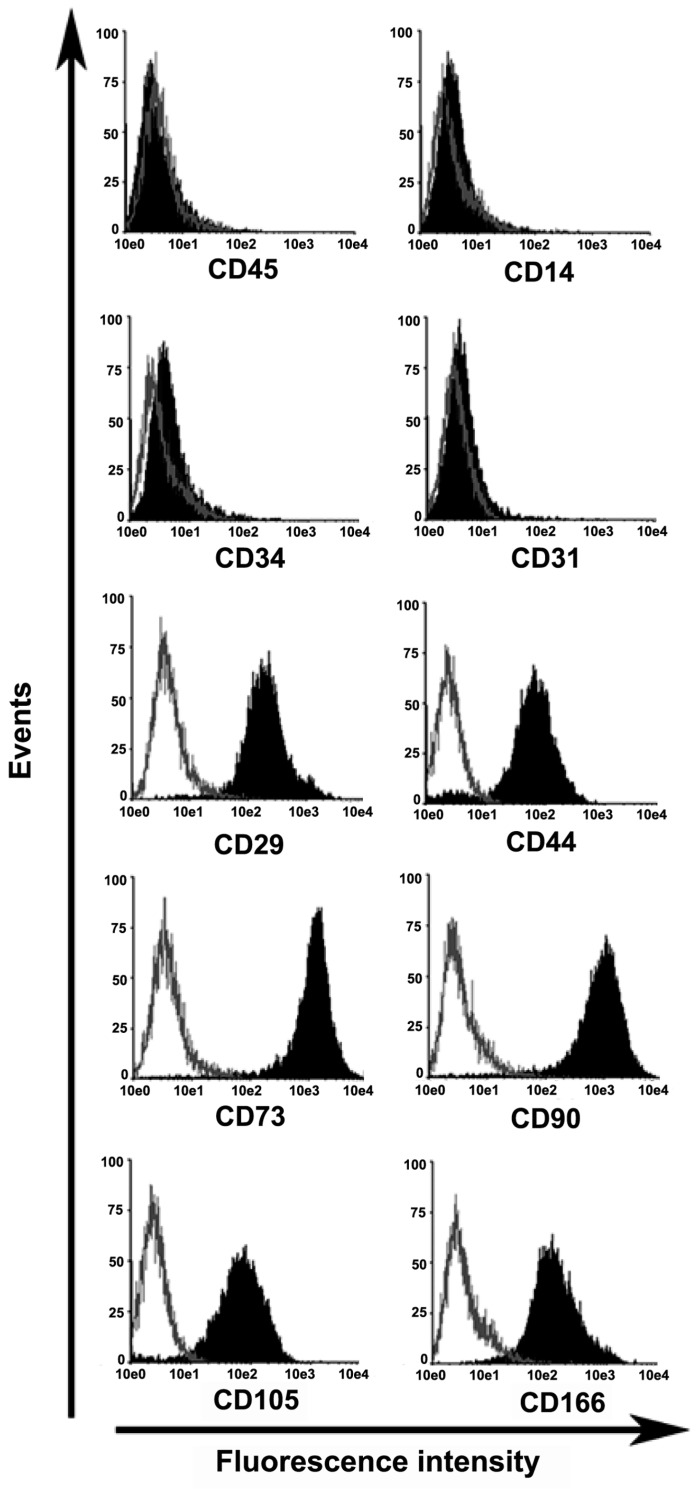
Characterization of umbilical cord Wharton’s jelly (WJ)-derived mesenchymal stem cells (uMSCs). The cells were cultured as described in Section 2.3. As shown in the figure, flow cytometric experiments demonstrated that the WJ-derived cells expressed a panel of markers typical for MSCs, but not for hematopoietic or endothelial lineages. The results are shown for cells obtained from six umbilical cords, and the shape and position of the overall profiles shown here were consistent with those of the individual profiles for cells obtained from passages 5–8.

After 5–7 days in culture, spindle-shaped, plastic-adherent cells were observed proliferating from the Wharton’s jelly (WJ) explanted tissue. The cells completely covered the available growth area within 12 to 15 days. Umbilical cord WJ contains mucoid connective tissue and fibroblast-like cells. To confirm that the adherent cells derived from the WJ had MSC potential, the presence of surface markers associated with human MSCs was assessed [27,28,29]. Flow cytometric analyses demonstrated that the WJ-derived cells had the typical mesenchymal pattern of surface markers, i.e., CD29 (99%), CD44 (95%), CD73 (99%), CD90 (99%), CD105 (91%), and CD166 (90%), but did not have hematopoietic or endothelial-type markers, i.e., CD14 (< 1%), CD34 (< 2.5%), CD45 (< 1%), and CD31 (< 1%) (Figure 1). These observations indicated that these WJ-derived cells (uMSCs) had MSC potential.

### 2.2. Differentiation of uMSCs into Myoblast-Like Cells In Vitro

**Figure 2:**
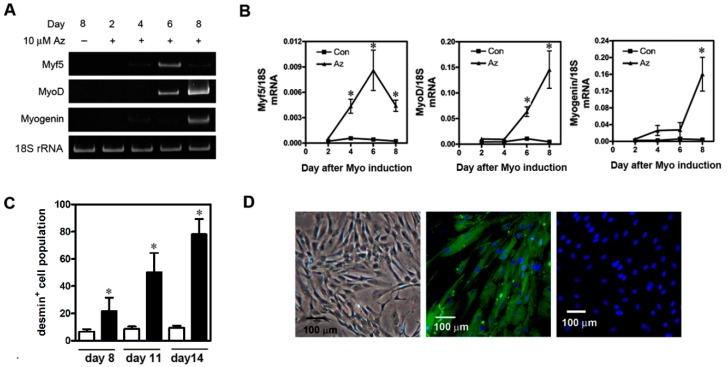
In vitro differentiation of uMSCs into myoblast-like cells. The uMSCs were treated with 10 μM 5-azacytidine and then cultured for two, four, six, or eight days in myogenic inductive medium, after which the expression of the myogenic-lineage genes for myogenic factor 5 (Myf5), myogenic determination factor (MyoD), and myogenin was determined by (**A**) RT-PCR and (**B**) quantitative PCR. Cells cultured in Dulbecco’s Modified Eagle Medium (DMEM) (high glucose) supplemented with 20% Fetal Bovine Serum (FBS) served as negative controls. Data are shown as means ± standard deviation (SD) of six independent experiments using different batches of cells and were analyzed by ANOVA with repeated measurement. (**C**) The percentage of desmin^+^ cells present on days eight, 11, and 14 after uMSCs were myoinduced to differentiate in vitro. To quantify the number of desmin^+^ cells present, the cells were firstly stained with an anti-desmin antibody and an isotype-matched Fluorescein isothiocyanate (FITC)-Immunoglobulin (Ig)G, and then quantified using flow cytometry. The data for each time point were obtained from four independent experiments, reported as means ± SD. (**D**) Representative images of uMSCs grown in vitro before and after myoinduction. Left panel: undifferentiated uMSCs had a spindle-like appearance. Middle panel: desmin^+^ cells 14 days after myoinduction that were identified using an anti-desmin antibody as described above. Right panel: negative controls that did not include the anti-desmin antibody. Scale bar, 100 μm; * *p* < 0.05 for induced vs. uninduced cells.

The differentiation of mesenchymal stem cells into myogenic cells is associated with the early expression of muscle-specific transcription factors, myogenic factor 5 (Myf5), myogenic determination factor (MyoD), and myogenin [21,30,31]. The expression of these factors is followed temporally by the expression of desmin, a muscle-specific intermediate filament protein, which is upregulated during musculoskeletal myogenesis. We performed quantitative PCR to investigate whether the cells could express these myogenic markers and could undergo myogenic differentiation in vitro (Figure 2A,B). The expression of Myf5 mRNA was measurable by day four following 10 μM 5-azacytidine administration, reached a maximum on day six, and then gradually declined until the experiment was ended on day eight. MyoD mRNA expression increased linearly from day four to day eight. Substantial expression of myogenin mRNA was found on day eight. Conversely, expression of the corresponding mRNAs did not occur in undifferentiated, control uMSCs (Figure 2A).

We also quantified the desmin^+^ cell population after myoinduction using flow cytometry. We determined that, after induction, the desmin^+^ cell population increased in a linear manner beginning on day eight (21.60% ± 9.9%) and reached a maximum on day 14 (78.13% ± 11.19%) when the experiment was ended (Figure 2C). On the other hand, the undifferentiated uMSCs did not synthesize desmin. Morphologically, the undifferentiated uMSCs were fibroblast-like in appearance (Figure 2D, left panel), whereas, 14 days after myoinduction, the treated cells became myoblast-like (Figure 2D, middle panel). After immunocytochemical staining on day 14, desmin^+^ cells were observed (Figure 2D, middle panel). The reaction against anti-desmin antibodies was not observed in assays for which the antibody was omitted (Figure 2D, right panel) or in assays that used undifferentiated cells.

### 2.3. BPVC-Induced Functional Impairment of Gait Was Rescued after Transplantation of uMSCs

**Figure 3:**
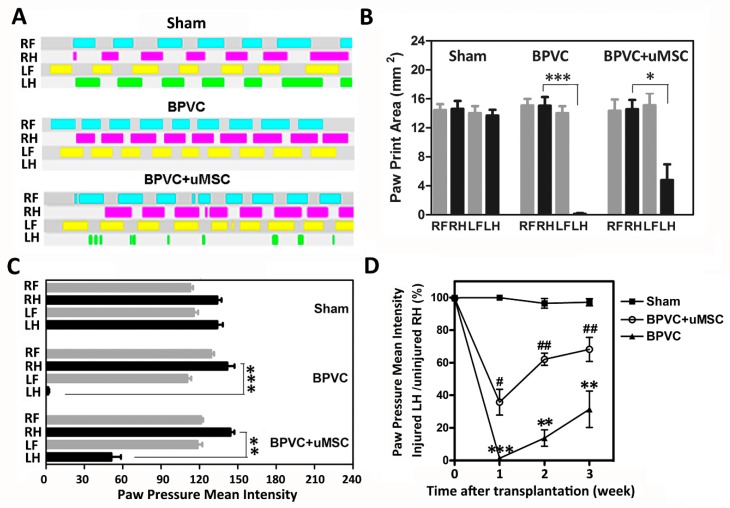
Bupivacaine hydrochloride (BPVC) induced quadriceps muscle necrotic injury and functional impairments, whereas transplantation of uMSCs mitigated the progressive loss of functionality of muscle in vivo. We injected 60 μL of 1.5% (*w*/*v*) BPVC into left hind (LH) limb quadriceps muscles of C57BL/6 mice to induce muscle necrosis (BPVC group). Injection of an equal volume of the saline into the right hind (RH) limbs quadriceps muscles served as the contralateral control. For mice in the sham control group (Sham), quadriceps muscle of both LH and RH limbs received a saline injection. Intramuscular injections of 5 × 10^5^ uMSCs in 60 μL of saline were performed one day post BPVC injury (BPVC + uMSC). (**A**) Representative picture of the CatWalk gait diagram three days after saline injection and BPVC injection, as well as three days after uMSC injection post BPVC injury. Indicated are the paw–floor contacts for each of the paws over time. (RF, right front paw; RH, right hind paw; LF, left front paw; LH, left hind paw). Assessment of gait parameters using the CatWalk system, including (**B**) paw print area and (**C**) paw pressure mean intensity as a measure of functional impairment after three days of saline or BPVC injection, and three days of uMSC injection post BPVC injury. Data are reported as means ± SD; ** *p* < 0.01, *** *p* < 0.001 for BPVC-injured LH limb vs. the contralateral uninjured control RH limb (*n* = 6 mice/group). (**D**) Progressive paw pressure mean intensities of the injured LH limbs of the three groups were recorded one, two, and three weeks later. The value of intensity for a mouse’s LH paw was normalized to the intensity value (100%) of its contralateral RH paw control (*n* = 6 mice/group); * *p* < 0.05, ** *p* < 0.01, *** *p* < 0.001 for BPVC group vs. sham group; # *p* < 0.05, ## *p* < 0.01 for BPVC + uMSC group vs. BPVC group.

Next, to evaluate whether uMSCs exerted a therapeutic effect on repairing muscle injury in vivo, firstly, we established a quadriceps muscle-injured model via injection of myotoxin bupivacaine hydrochloride (BPVC) in male C57BL/6 (B6) mice [25,26]. BPVC interacts with a calcium release channel ryanodine receptor at the membranes of the sarcoplasmic reticulum to increase intracellular calcium levels, in turn activating muscle proteases, leading to the death of muscle fibers. Mice were randomly assigned into three experimental groups of six animals each. In the BPVC-injured group, we intramuscularly injected the solution of BPVC (60 μL of 1.5% (*w*/*v*) in 0.9% saline solution) into the LH limb quadriceps muscles of C57BL/6 mice to induce muscle injury, and injected an equal volume of saline into their RH limbs to serve as the contralateral control as described in Section 2.5. In the BPVC injury combined with uMSC group, after one day of BPVC injury, undifferentiated uMSCs (5 × 10^5^) were injected into quadriceps muscles. For mice in the sham control group, quadriceps muscles of both LH and RH limbs received a saline injection. The gait function of the hind limbs was evaluated using the CatWalk assay as described in Section 4.9 to record the paw-floor contacts and related gait parameters for each of the paws over time. Remarkably, after BPVC treatment, the early impairment of gait was shown, and mice limped noticeably on their injured LH limbs. Figure 3A shows the modest impairment of gait by the CatWalk gait diagram three days after LH limbs were injured with BPVC, compared to their contralateral control RH limbs or the sham control group. After quantification, we observed significant reduced gait parameters, including paw–floor print areas (Figure 3B) and paw pressure mean intensity (Figure 3C), of LH limbs apparent in BPVC-injured group. Noteworthy, functional impairments (both of the paw-floor print areas and paw pressure mean intensity) were effectively rescued three days post uMSC transplantation (Figure 3B,C). To address the longitudinal contribution of uMSC transplantation to the recovery of functional impairment of gait post BPVC injury, paw pressure intensity was consecutively recorded on weeks one, two, and three for the three groups. Consistently, we observed that the ratio of paw pressure mean intensity (injured LH limbs normalized to their contralateral uninjured RH limbs) markedly dropped post BPVC injury, but gradually recovered in a time-dependent manner. Transplantation of uMSCs successfully extricated BPVC-impaired gait function at all three time points.

### 2.4. Attenuation of BPVC-Induced Neutrophil Infiltration and Chemotaxis of Neutrophil Recruitment/Activation in Quadriceps Muscles after uMSC Transplantation

**Figure 4:**
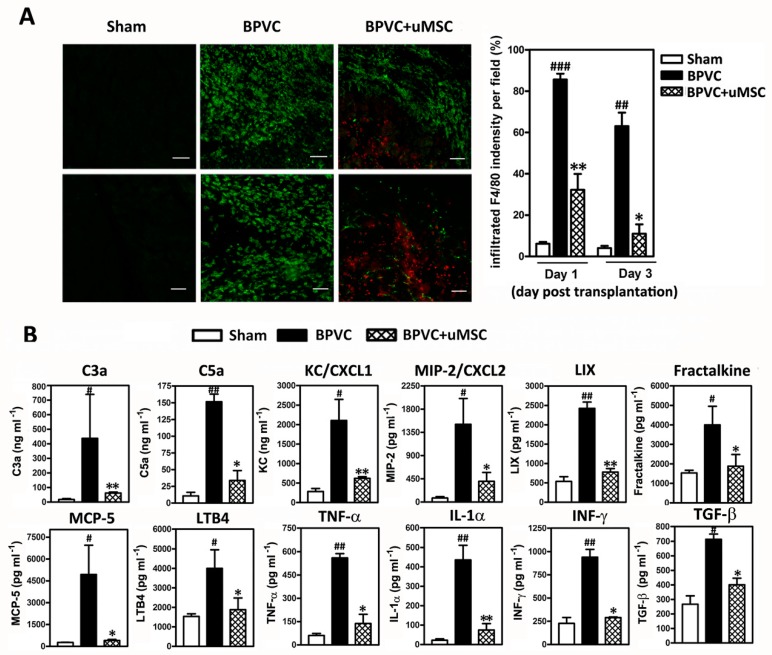
The uMSCs attenuated the BPVC-induced neutrophil infiltration and activation and ameliorated the elevated chemotaxis of cytokines in the early-onset inflammation. (**A**) Representative histological images of tissue sections that show the extent of neutrophil infiltration after BPVC injection. The necrotic regions of the quadriceps muscles two days (upper three panels) or four days (lower three panels) after BPVC injection (middle panel) had elevated neutrophil infiltration (mouse neutrophils were detected by immunolocalization of mouse Lymphocyte antigen 6 complex locus G6D (Ly6G) staining, shown in green fluorescence). The uMSCs were transplanted 24 h after BPVC-injection. Neutrophil infiltration was markedly attenuated following one or three days of uMSC transplantation (right panel; transplanted human uMSCs were localized by immunostaining with a human-specific ribonuclear protein antibody [32,33], presented in red fluorescence). Histograms show the quantification of infiltrated mouse Ly6G per field (percentage ratio) from quadriceps muscles isolated from C57BL/6 mice which underwent saline-or BPVC injection, or transplantation of uMSCs post BPVC injury. Scale bar: 100 μm. (**B**) Tissue extracts were collected from quadriceps muscles on day four post saline or BPVC injection, or after three days of uMSC transplantation 24 h following BPVC-injection. The protein levels of chemotaxis for neutrophil activation and recruitment or released cytokines (pro-inflammation or pro-fibrosis) associated with each group of mice were determined using the Quantibody Mouse Cytokine Array (*n* = 6/group). Data are given as means ± SD; # *p* < 0.05, ## *p* < 0.01, ### *p* < 0.001 for BPVC group vs. sham group; * *p* < 0.05, ** *p* < 0.01 for BPVC + uMSC group vs. BPVC group.

After BPVC injection, we observed a robust inflammatory infiltration surrounding the necrotic fibers, and we found that the inflammatory infiltrate was composed of Ly6G-FITC^+^ neutrophils by immunofluorescence staining (Figure 4A, middle panel; green fluorescence). At 24 h post BPVC injection, during the peak of neutrophil infiltration, we immediately transplanted uMSCs into the injured quadriceps muscles. To ensure that the transplanted human uMSCs really presented in the necrotic quadriceps muscle site, tissue specimens were co-stained with human nucleus antibody [32,33]. (for human uMSCs; Figure 4A, right panel; uMSCs are shown in red fluorescence and were detected within the injured quadriceps muscles). We observed that the transplantation of uMSCs remarkably eliminated the BPVC injury-induced Ly6G-FITC^+^ neutrophil infiltration (about 63.1% ± 5.2% and 83.6% ± 3.8% reduction rate after one and three days of uMSC transplantation, respectively, compared to the BPVC injury group).

Next, to illustrate the mechanisms responsible for the attenuation of the BPVC injury-induced neutrophil infiltration by the uMSCs, we estimated the levels of chemokines for neutrophil recruitment or activation, as well as the neutrophils released during chemotaxis using a Quantibody Mouse Cytokine Array (Raybiotech Inc) as described previously [29]. and quantified the protein levels obtained from the quadriceps muscles of LH limbs of three groups. As shown in Figure 4B, we innovatively clarified a portion of the elevated level of chemokines to recruit peripheral neutrophils [Keratinocyte chemoattractant (KC), Macrophage Inflammatory Protein 2 (MIP-2), LPS-induced CXC chemokine(LIX), Fractalkine, Leukotriene B4 (LTB4), monocyte chemotactic protein (MCP)-5)], chemokines to activate resident neutrophils [complement-derived chemotactic factor; C3a and C5a], and released pro-inflammatory cytokines [Tumor necrosis factor (TNF)-*α*, Interferon (IFN)-*γ*, and Interleukin (IL)-1α] in BPVC-damaged quadriceps muscles tissue (Figure 4B). Transplantation of uMSCs notably abridged neutrophil infiltration and attenuated chemotaxis for neutrophil recruitment and activation. Our data provided a reasonable basis for the invasion of neutrophils into the injured site and strikingly supported the uMSCs contributing an anti-inflammatory role in BPVC-induced quadriceps muscle injury through eliminating neutrophil-derived acute inflammation.

### 2.5. Abrogation of BPVC-Induced Quadriceps Muscle Fibrosis after uMSC Transplantation via Alleviating TGF-β-triggered Canonical Smad2-Dependent and Non-Canonical Smad-Independent TAK1/p38 Signaling Pathways

**Figure 5:**
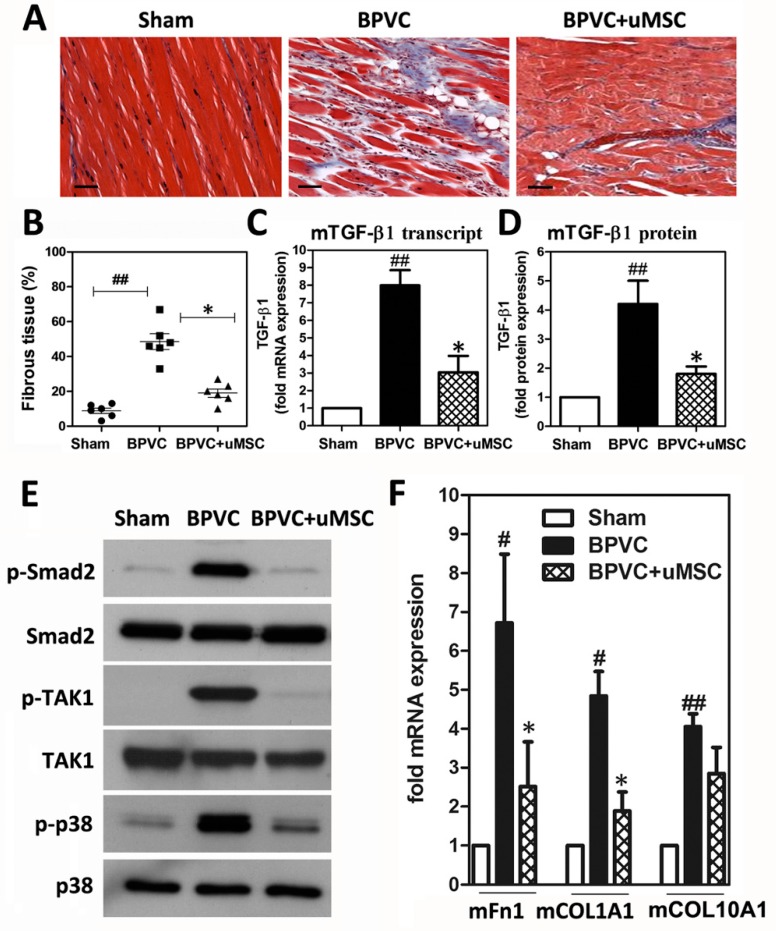
The presence of uMSCs abrogated BPVC-induced fibrosis. Quadriceps muscles were isolated from three groups of C57BL/6 mice after two weeks of BPVC injection or uMSC transplantation. (**A**) Representative immunohistochemistry figures of quadriceps muscles with the nuclei stained black, the muscle cells stained red, and fibrous tissue stained blue by Masson’s trichrome staining. At least five different tissue sections from each group of mice were examined, and their appearances were similar (*n* = 6/group). Scale bar: 100 μm. (**B**) The extent of fibrosis was scored after Masson’s trichrome staining on day 14 post uMSC transplantation. The level of fibrosis was significantly reduced after 14 days of uMSC transplantation. (**C**) The transcript levels of mouse Transforming Growth Factor (TGF)-β1 were determined using quantitative PCR (*n* = 6). For the transcript levels determined using messenger RNA (mRNA) extracted from the LH limb quadriceps muscles, the values were each firstly normalized to that of the Glyceraldehyde 3-phosphate dehydrogenase (GAPDH) internal control and then to the value obtained using mRNAs extracted from the quadriceps muscles of the sham control. (**D**) The enzyme-linked immunosorbent assay for mouse TGF-β1 demonstrated a significant increase in TGF-β1 on day 14 post BPVC injection with a significant reduction in levels by uMSC transplantation (*n* = 6). (**E**) Representative image of the Western blot analysis for phospho- Sma- And Mad-Related Protein (Smad)2 (Ser465/467), a transcription factor mediating the canonical signaling of TGF-β1, showing an increase on day 14 post BPVC injury and a decrease with uMSC transplantation (*n* = 6). Meanwhile, TGF-β1 regulated noncanonical signaling, phosphorylation of Transforming growth factor beta-activated kinase (TAK)1 and p38, which was also elevated in the BPVC-injured groups, and transplantation of uMSCs noticeably diminished the phospho-TAK1 and phospho-p38 levels (*n* = 6). (**F**) Transplantation of uMSCs on day 14 post BPVC injury also reduced transcription levels of BPVC injury-induced extracellular matrix (ECM) genes including fibronectin (Fn1), collagen (COL) 1A1, and COL10A1, which are regulated by both Smad2/3 and p38. Data are given as means ± SD; # *p* < 0.05, ## *p* < 0.01 for BPVC group vs/ sham group; * *p* < 0.05 for BPVC + uMSC group vs. BPVC group.

It was shown that the development of fibrotic tissue is one of the major factors affecting the recovery of muscle function post injury [34]. Notably, after three days of BPVC injection, TGF-β1 and IFN-γ, as well as known pro-fibrotic cytokines released by neutrophils, were significantly elevated in the injured muscle (Figure 4B), and one day or three days of engraftment of uMSCs dramatically reduced those levels after transplantation. Therefore, we subsequently analyzed the effects of uMSCs on the development of fibrosis, which immediately followed the inflammatory stage. Paraffin sections of the quadriceps muscles from all three groups were stained with the reagents of a Masson trichrome staining kit so that the relative amounts of fibrous tissue in the quadriceps muscles could be evaluated (Figure 5A). We observed that, two weeks post injury, there was a significant increase in the amounts of fibrotic tissue due to BPVC administration present in the quadriceps muscles compared to the saline-injected quadriceps muscles (sham control group, 14.5% ± 1.61%; BPVC-injured group, 43.83% ± 5.67%) (Figure 5B). Noteworthy, mice which received two weeks of uMSC transplantation had markedly reduced 55.74% ± 4.88% percentage ratios in BPVC injury-induced amounts of fibrotic tissue (Figure 5B).

To explore how the uMSCs reduced BPVC-induced fibrosis, using quantitative PCR and ELISA, we quantified the expression level of TGF-β1, the predominant isoform of the TGF-β superfamily, which mediates tissue fibrosis associated with inflammation and tissue injury. After two weeks of BPVC treatment, TGF-β1 transcription and protein expression were significantly enhanced (7.83 ± 0.71-fold and 4.07 ± 0.82-fold increases compared to sham group, respectively) in the quadriceps muscles (Figure 5C), whereas uMSC transplantation effectively abrogated the BPVC-induced expression of TGF-β1 transcription and protein expression (about 67.9% ± 2.8% and 53.6% ± 4.1% reduction rates compared to BPVC injury group, respectively) (Figure 5D). The TGF-β1 protein levels of mouse muscle tissue nicely mirrored their transcription pattern (Figure 5C,D). Canonical TGF-β signaling mobilizes Smad2/3 transcription factors that control fibrosis by promoting extracellular matrix protein expression [35]. Interestingly, we observed that phospho-Smad2 (Ser465/467) was upregulated by BPVC injection and manifestly abrogated by uMSC transplantation (Figure 5E). Of note, we also observed a BPVC-induced increase in TGF-β activating kinase 1 (TAK1, also known as Mitogen-activated protein kinase kinase kinase 7 (MAP3K7) and p38 mitogen-activated protein kinases phosphorylation, involved in the TGF-β-activated noncanonical signaling MAPK cascade [35], in the injured quadriceps muscles. Transplantation of uMSCs efficiently attenuated the BPVC-enhanced phospho-TAK1 and phospho-p38 levels. Herein, we provide innovative evidence to illustrate that the administration of BPVC may activate the master regulator TGF-β to enhance tissue fibrosis, which subsequently transmits its signal via the canonical Smad2-dependent and non-canonical Smad-independent signaling pathways, including phospho-TAK1 and phospho-p38 activation. Furthermore, we identified transcript levels of three important extracellular matrix components including fibronectin (Fn1), collagen (COL)1A1, and COL10A1 (Figure 5F), genes which are known to regulated by both Smad-2/3 and p38 [36,37,38], and which were modestly enhanced post BPVC injury. In sum, transplantation of uMSC effectively alleviated the BPVC-enhanced quadriceps muscle fibrosis via diminishing TGF-β expression levels, as well as the TGF-β triggered activation of various downstream signaling cascades.

## 3. Discussion

For the study reported herein, we established a skeletal muscle injury model via injection of myotoxin BPVC into quadriceps muscles of C57BL/6 mice. We found that, after BPVC injection, neutrophils rapidly invaded injured muscle tissue post injury. Transplantation of uMSCs remarkably abolished neutrophil infiltration and activation, and mitigated the robust elevation of chemotactic cytokines modulating the neutrophil chemotaxis, indicating the immune-modulatory role of uMSCs in ameliorating neutrophil-mediated acute inflammation post injury. Subsequently, elevated collagen deposition was shown in injured quadriceps muscles tissue after two weeks of BPVC injection, whereas engrafted uMSCs exhibited an anti-fibrosis role to protect against the fibrous tissue formation post injury. Transplanted uMSCs abridged the fibrosis, potentially through interrupting the canonical TGF-β1/Smad2/3 pathway and noncanonical TGF-β1/TAK1/p38 signaling, which in turn reduced the expression of extracellular matrix component genes Fn-1, COL1A1, and COL10A1. Notably, engrafted uMSCs modestly improved BPVC-impaired gait functions. This work provides various novel insights and a molecular basis for the early elimination of neutrophil-mediated inflammation by uMSCs, which diminished the intensity of events that occur at a later time, e.g., fibrous tissue formation, thereby allowing for an improvement in functional recovery.

Numerous studies demonstrated that MSCs have the potential to differentiate into myoblast-like cells in vitro and in vivo [20,21]. The uMSCs used in this study was able to differentiate into a myogenic lineage in vitro and subsequently expressed numerous muscle-differentiation markers, Myf5 (peak at six days post induction), MyoD (peak at eight days post induction), myogenin (expressed at eight days post induction), and desmin (peak at two weeks post induction) (Figure 2A–D). This sequential time-dependent pattern of myogenic gene expression is similar to that found during mouse embryo development and for cultured human myoblasts [21,39]. Our data are also consistent with a previous report by Conconi et al [21]. They demonstrated that stem cells derived from WJ could differentiate in vitro into myoblast-like cells, expressing My5 and MyoD after seven and 11 days of myogenic induction [21]. Similar, we also could not detect the sarcometric myosin heavy-chain protein, which is a marker for mature muscle fibers, in our in vitro system. These observations are consistent with previous reports which found that, when a network of nerves and/or hormones was not present, or when there was insufficient cell-to-cell contact in vitro, incomplete myogenic differentiation occurred [40,41]. Additionally, a previous study reported the injection of myoblast-like cells from an expanded primary culture into severely damaged skeletal muscles [42]. However, various therapeutic limitations of myoblast transplantation were shown, including poor cell survival and limited migratory ability of cells at the injury site, hindering the application of this therapy [42]. Fortunately, other studies demonstrated that intravenous injection of undifferentiated bone-marrow-derived MSCs successfully migrated to the injury site and improved mouse skeletal muscle healing [43,44]. Recently, the injection of isolated HUCB MSCs into the quadriceps of mdx (mouse dystrophy X-chromosome-linked) mice was also demonstrated to locally contribute dystrophin, which enhanced muscle regeneration [21,45]. Herein, we demonstrated that the injection of undifferentiated umbilical cord Wharton’s jelly MSCs into BPVC-injured quadriceps muscles noticeably exhibited an immune-modulatory role to attenuate acute inflammation, decrease fibrosis, and enhance functional recovery.

Accumulating evidence suggests that inflammatory cells are involved in muscle repair ^4^. After skeletal muscle injury, the injured muscle cells undergo necrosis in response to trauma. Neutrophils are the most prevalent innate immune cells. After being released into the blood, neutrophils guard the circulatory system, protecting the host from pathogens until they encounter inflammatory signals. Upon muscle injury, the membranes of muscle fibers are damaged, and cellular contents ^4^ and complement-derived chemotactic factors [46] are released from the leakage of fibers into the extracellular space, leading to immediate activation of the resident neutrophils in skeletal muscles post injury. Herein, we focused on exploring whether and how engrafted uMSCs influence neutrophil expression, activation, and chemotaxis in early-stage inflammation. We found a fast infiltration of neutrophils (> 90% per field) in the quadriceps muscles 24 h after BPVC injection, which persisted for at least 72 h (around 60% per field) post injury (Figure 4A), which is in agreement with previous studies [26,47]. Additionally, we innovatively clarified a large portion of chemokines for recruiting peripheral neutrophils, chemokines for activating neutrophils, and released pro-inflammatory cytokines, elevated in BPVC-damaged quadriceps muscles tissue (Figure 4B). Transplantation of uMSCs during the peak of neutrophil infiltration post BPVC injury modestly reduced neutrophil invasion and released chemotaxis (Figure 4B). Our data provide support for the invasion of neutrophils into the injured site, and engrafted uMSCs effectively abridged neutrophil recruitment and activation, thus ameliorating early-onset inflammation. Previous studies demonstrated that the anti-inflammatory role of uMSCs may act through the suppression of dendritic cell maturation or the inhibition of T-cell and B-cell proliferation [48,49]. Herein, we suggest a novel immunomodulatory role of uMSCs in the regulation of neutrophils via ameliorating neutrophil invasive burst and activation, which in turn limits early-onset inflammation in vivo.

We next explored whether uMSC-abrogated neutrophil invasion during early-onset inflammation may contribute to regulating the muscle injury process. Neutrophils are reported to display a crucial role in acute inflammation through the removal of necrotic tissue or cellular debris and the release of cytokines to modulate chemotaxis [4]. However, during the process of neutrophils destroying damaged tissues, neutrophils can also undergo a respiratory burst to generate hypochlorous acid via an myeloperoxidase (MPO)-mediated reaction, as well as superoxide via NAD phosphate (NADPH) oxidase, which can potentially contribute to worsening the muscle damage during injury [50]. In agreement with this scenario, accumulated studies provided evidence that neutrophil infiltration may lead to “secondary injury”, exacerbating the muscular damage during the early inflammatory period [24]. For example, Brickson et al. reported that peak neutrophil infiltration of the damaged muscle occurs within 24 h after stretch injury and is associated with both maximum fiber tearing and maximum oxidant production [51]. Another important observation from St. Pierre Schneider et al. defined both the degree of muscle fiber damage and the neutrophil infiltration of stretch-injured muscle increase at 24 h post injury [52]. Furthermore, Korthuis et al. demonstrated that neutrophils may increase muscle fiber degradation through increased neutrophil-derived reactive oxygen species using an ischemia–reperfusion (I/R) injury model [23]. Recently, MSCs were found to inhibit the production of intracellular hydrogen peroxide levels via co-culture with neutrophils in vitro [53]. We also recently demonstrated that transplantation of human umbilical cord MSCs recaptured impaired oxidative phosphorylation (OXPHOS) and bioenergetics caused by mitochondrial DNA defects [28,29]. This evidence suggests the oxidative suppression role of MSCs. Accordingly, we presumed that employing uMSCs can limit neutrophil-mediated acute inflammation and at least potentially attenuate the intensity of respiratory burst or oxidative stress upon inflammatory stimulation, thus delivering benefits in protecting against secondary damage post muscle injury. On the other hand, in our study, we injected a dose of 1.5% (*w*/*v*) BPVC into quadriceps muscles to establish a skeletal ischemic muscle injury model of C57BL/6 mice [25]. Doses of 1.5% or 1% (*w*/*v*) BPVC injection were demonstrated to produce significant levels of ischemic muscle fibers post injury [25,26]. After BPVC injection, we observed that neutrophil infiltration was immediately detected and peaked around 24 h post injury. Therefore, after 24 h of BPVC-injection, during the peak of neutrophil infiltration, we transplanted uMSCs into the injured quadriceps muscles. Engrafted uMSCs were able to strikingly abrogate the neutrophil infiltration (Figure 4A). Noticeably, we further observed that the uMSC-abrogated early onset of neutrophil invasion markedly reduced subsequent fibrosis (over 50% reduction in fibrous tissue) at two weeks post injury (Figure 5A,B). Our observation mirrored the findings of Korthuis et al. who reported that neutrophil depletion before ischemia–reperfusion (I/R) skeletal muscle injury can attenuate histologically detectable damage by up to 40% [23]. Additionally, we also found that engrafting uMSCs intensely abridged neutrophil-released pro-fibrotic cytokines TGF-β1 and IFN-γ in the injured muscle, three days after BPVC injection (Figure 4B). Our finding is also consistent with the report of Warren et al. [54], who showed that the first peak expression of TGF-β1 occurs two to three days after muscle injury and can promote fibrosis at the injured site. Furthermore, Ota et al. reported that the injection of muscle-derived stem cells at four or seven days post injury minimized fibrosis; of note, muscle strength improved only with stem cell injection at the earlier time point [55]. Thus, our findings provide reasonable rationale for why the earlier stage of uMSC transplantation noticeably diminished the fibrous tissue formation observed at two weeks post injury (Figure 5A,B), leading to functional recovery (Figure 3). This likely occurred through a mechanism to decrease neutrophil-released pro-fibrosis cytokines by uMSCs in early-onset inflammatory phase. Our data provide evidence that engrafted uMSCs at 24 h post injury were sufficient to protect against the excessive activation of neutrophils, thus attenuating the inflammatory network; this was beneficial for later muscle healing, especially protecting against fibrous tissue formation and functional recovery.

The formation of fibrotic tissue results in inadequate healing and deficient muscle function. TGF-β is the key cytokine involved in the pathogenesis of muscle healing [56]. In particular, in skeletal muscle, it inhibits myogenic responses, regulates extracellular matrix (ECM) remodeling, and stimulates fibrosis [35,57]. For muscle diseases characterized by fibrosis, such as muscular dystrophy and inflammatory myopathy, TGF-β is localized to the extracellular matrix between the myofibers and the areas of inflammatory-cell infiltration [57]. Several studies demonstrated that blockage of TGF-β action improves muscle regeneration and functional recovery from injury by decreasing the levels of factors related to muscle damage, such as creatine kinase and fibrosis [58]. TGF-β exists as three subtypes, namely, TGF-β1, TGF-β2, and TGF-β3 [35]. Among these, TGF-β1 represents the predominant isoform of the TGF-β superfamily and mediates tissue fibrosis associated with inflammation and tissue injury [35]. Our data demonstrated that the anti-fibrotic role of uMSCs was reflected in the reduced TGF-β1 levels both at an earlier stage (day three post uMSC injection; Figure 4B) and later stage (day 14 post uMSC injection; Figure 5C,D). We also observed a significant reduction in histological fibrosis score in BPVC-injured tissue after two weeks of uMSC transplantation (Figure 5A,B). These findings suggest that uMSCs may decrease fibrosis by eliminating levels of the critical pro-fibrotic cytokine TGF-β1. We next aimed to clarify the signaling cascades of TGF-β1 in response to uMSC-mediated anti-fibrosis. TGF-β1 signals through heterodimeric type I and II receptors (TβRI and TβRII), members of the serine/threonine kinase family. In the canonical pathway, the TβRI and TβRII complex activates Smad transcription factors (mainly through Smad2/3 phosphorylation) [59]. Activation of Smad2/3 regulates the expression of several pro-fibrotic genes, including various collagens (*COL1A1, COL3A1, COL5A2, COL6A1, COL6A3, COL7A1,* and *COL10A1*) [60], proteoglycans [61], and integrins [62]. In agreement with these findings, we observed that phospho-Smad2 (Ser465/467) was upregulated by BPVC injection and noticeably abrogated by uMSC transplantation (Figure 5E). Hence, TGF-β1-mediated noncanonical signaling through TβRI/TβRIII complex activation leads to TAK1/p38 phosphorylation, as also reported in the prevention of congenital craniofacial birth defects [63], and in the protection of ventricular hypertrophy and post-myocardial infarction remodeling in rats [64]. Of interest, we also observed a Smad-independent noncanonical signaling pathway through phosphorylation of TAK1 and p38, activated by BPVC injection (Figure 5E). However, based on our knowledge, these finding regarding the uMSC-mediated signaling cascade to renovate BPVC-impaired skeletal muscle injury is novel and was never previously reported. Taken together, in skeletal muscle injury, we innovatively defined that transplanted uMSCs may abrogate TGF-β1-mediated signaling cascades, including canonical Smad2 activation and noncanonical TAK1/p38 signaling, to block BPVC injury-induced fibrosis-related ECM genes, including Fn1, COL1A1, and COL10A1 production, in the quadriceps muscles of C57BL/6 mice.

## 4. Materials and Methods

### 4.1. Chemicals and Antibodies

Anti-human CD antigen (CD)29, CD73, CD44, CD90, CD166, CD105, CD31, CD14, CD34, and CD45 antibodies were purchased from BD Biosciences (San Jose, CA, USA). Transplanted uMSCs were identified using a human-specific ribonuclear protein antibody (antibody 1281 to human nuclei), which was purchased from (Chemicon, Temecula, CA, USA) [32,33]. Anti-mouse Lymphocyte antigen 6 complex locus G6D (Ly6G), anti-mouse phospho- Sma- And Mad-Related Protein (Smad)2 (Ser465/467), anti-mouse Smad2, anti-mouse Transforming growth factor beta-activated kinase (TAK)-1, anti-mouse phospho-TAK1 (Ser412), anti-mouse p38, and anti-mouse phospho-p38 (Thr180/Tyr182) antibodies were obtained from Cell Signaling Technologies (Beverly, MA, USA).

### 4.2. Animals

Male C57BL/6 (B6) mice were purchased from the National Animal Center, Taiwan, and were maintained on a standard diet of chow and water that was made available ad libitum. The mice were housed in an animal facility illuminated between the hours of 6:00 a.m. and 6:00 p.m. All animal care procedures and experimental protocols used in this study were reviewed and approved by the Ethics Committee on Animal Experiments of Chang Gung Memorial Hospital, Kaohsiung Medical Center in Taiwan (CGMH; IACUC number 102-420C).

### 4.3. Isolation, Cultivation, and Characterization of Human uMSCs

The preparation of human uMSCs was described in our previous studies [28,29]. Human uMSCs were isolated from fresh human umbilical cords obtained during normal spontaneous deliveries after written informed consent was obtained. In brief, human umbilical cords were placed in Hanks’ balanced salt solution (Gibco, Carlsbad, CA, USA) before harvesting of the uMSCs. After the arteries and veins were removed, the remaining cord was diced into small pieces and transferred to 10-cm dishes containing Dulbecco’s Modified Eagle Medium (DMEM) in a 5% CO_2_, humidified atmosphere at 37 °C. Upon reaching 100% confluence, cells were detached using 0.25% trypsin- Ethylenediaminetetraacetic acid (EDTA) (Gibco, Carlsbad, CA, USA). The uMSCs had a typical spindle-shaped appearance and were found to be positive for CD29, CD73, CD44, CD90, CD166, and CD105 (BD Biosciences, San Jose, CA) and negative for endothelial and hematopoietic markers CD31, CD14, CD34, and CD45 (BD Biosciences, San Jose, CA, USA) in flow cytometric analysis. Appropriate isotype antibodies were used to monitor for nonspecific staining. Staining was performed according to the manufacturer’s recommendations, and, for each marker, at least 10^4^ immunostained cells were analyzed using a FACSCalibur flow cytometer (Becton Dickinson, Mountain View, CA, USA), under the control of cellQUEST software (Becton Dickinson). The uMSCs used for the study were within five to eight passages. Likewise, this study protocol and written informed consent were reviewed and approved by the Institutional Review Board of Chang Gung Memorial Hospital (CGMH; IRB number 201601867B0C101).

### 4.4. In Vitro Myogenic Differentiation

In vitro myogenic differentiation was performed according to the manufacturer’s protocol ^21^. Briefly, at passages five to eight, the uMSCs (5 × 10^3^ cells/cm^2^) were placed onto two-well chamber slides coated with 1:10 diluted BD Matrigel (BD Biosciences Pharmingen, San Diego, CA, USA). The cells were propagated to 70%–80% confluence in the proliferative medium. The cells were then cultured in myogenic inductive medium, DMEM (high glucose; Sigma-Aldrich, St. Louis, MO, USA) supplemented with 20% Fetal Calf Serum (FCS), 2 mM l-glutamine, 1% Non-Essential Amino Acids (NEAA), 1% antibiotic–antimicotic solution, and 10 μM 5-azacytidine (Sigma-Aldrich), which induces myogenic differentiation. After a 48 h culture period in the presence of myogenic inductive medium, the medium was changed to myogenic proliferative medium: DMEM (high glucose), 20% Fetal Bovine Serum (FBS), 2 mM l-glutamine, 1% NEAA, 1% antibiotic–antimicotic solution, 10% horse serum, and 1% chick embryo extract (ICN Biomedicals, Irvine, CA, USA). Cells that served as negative controls were cultured in the proliferative medium deprived of differentiation factors. For immunofluorescence imaging, cultures were rinsed twice with Phosphate Buffered Saline (PBS), fixed with 3.7% buffered paraformaldehyde, permeabilized with 0.5% (*w*/*v*) Triton X-100 for 15 min, and incubated with antibodies against human desmin (1:100) (Abcam, Cambridge, UK) for 1 h, followed by incubation with Fluorescein isothiocyanate (FITC)-conjugated anti-rabbit Immunoglobulin G (IgG) (Molecular Probes, Invitrogen, CA, USA) for 30 min at room temperature in the dark. Nuclei were stained with Hoechst 33258 (Vector Laboratories, Burlingame, CA, USA). For negative controls, the primary antibody was omitted. To quantify the number of uMSCs that expressed desmin, trypsinized cells were washed twice with PBS and then incubated with mouse anti-human desmin as described above. FITC-conjugated anti-rabbit IgG served as an isotype control. Flow cytometric measurements were performed using the FACScan apparatus (Becton Dickinson).

### 4.5. Quadriceps Muscle Injury Induced by Bupivacaine Hydrochloride Injection and Human uMSCs Transplantation

To injure the quadriceps muscles, the animals were anesthetized with isoflurane, followed by 60 μL of 1.5% bupivacaine hydrochloride (Sigma-Aldrich) prepared in a 0.9% saline solution injected intramuscularly with a 29-gauge needle into the left hind (LH) limb quadriceps muscles of C57BL/6 mice at the ages of 10–12 weeks to induce ischemic muscle necrosis as previously described [26]. The quadriceps muscles of the other limb, right hind (RH) limb, was injected with 60 μL of 0.9% saline to serve as the contralateral control. In the BPVC injury combined with uMSC group, an intramuscular injection of undifferentiated human uMSCs (5 × 10^5^ cells) was administrated into the LH limb quadriceps muscles at 24 h post BPVC injury. The sham group received 60 μL of 0.9% saline injection into the quadriceps muscles of both the LH and RH limbs instead of BPVC treatment or uMSC transplantation. To delineate the role of human uMSCs in the regulation of BPVC-mediated inflammation, some of the animals in each group (*n* = 6) were sacrificed at two or four days after receiving BPVC or saline injections, whereas, for the BPVC injury combined with uMSC transplantation group (*n* = 6), animals were sacrificed at two or four days post BPVC injury (which means one or three days of uMSC transplantation). To investigate the role of human uMSCs in BPVC-induced fibrosis, the other three groups (*n* = 6 each) were sacrificed at two weeks after receiving BPVC injection, saline injection, or two weeks of uMSC transplantation post injury. After sacrifice, the animals were weighed, and the quadriceps muscles were removed, snap-frozen, and stored at −80 °C or immediately fixed in 10% formalin for paraffin embedding until analysis. Tissue sections were scored according to the extent of interstitial inflammatory-cell infiltration and fibrosis of the necrotic areas. After tissue sectioning, total RNA or total protein extracts were isolated from the remaining tissue for further analysis.

### 4.6. Cytokine Analysis by Quantibody Mouse Cytokine Array

Quadriceps muscle extracts were collected, and approximately 100 mg crude tissue was transferred into a tube with 1 mL of 1 × lysis buffer. The tissue was homogenized according to the manufacturer’s instructions [65]. Tissue extracts were transferred to microfuge tubes and placed in a centrifuge at top speed for 20 min (4 °C); supernatants were collected and stored at −80 °C. Before analysis, samples were centrifuged again at 13,000 *×g* for 5 min. The concentration of mouse TGF-β1 in quadriceps muscles was determined by enzyme-linked immunosorbent assay (ELISA) according to the manufacturer’s protocol (R&D Systems, Minneapolis, MN, USA). Chemokine/cytokine levels in quadriceps muscles were estimated using the Quantibody^®^ Mouse Cytokine Array according to the manufacturer’s protocol (Raybiotech Inc, Norcross, GA, USA) [65].

### 4.7. Muscle Fibrosis

Paraffin sections (5–7 μm thick) from each hind limb of each mouse were stained with the reagents of a Masson trichrome staining kit (Sigma-Aldrich) according to the manufacturer’s specifications [66]. Five high-powered image fields within a sectioned tissue were selected randomly, and the images were analyzed using image analysis software (Image-pro plus 4.0, Media Cybernetics LP, USA) to measure the area of fibrous tissue (blue-stained tissue) within the section [67]. The fibrotic area was expressed as the percentage of the entire cross-sectional area of the tissue sample. A blinded observer performed all of the analyses.

### 4.8. Reverse Transcription and Quantitative PCR

Procedures for RNA isolation, concentration, quality determination, RT-PCR, and quantitative PCR were described elsewhere [68]. Briefly, RNA was extracted using TRI Reagent (Sigma-Aldrich), treated with DNase I (Ambion, Austin, TX, USA), and reverse-transcribed with random primers (Invitrogen). A negative control that omitted the reverse transcriptase was always performed to ensure that the messenger RNA (mRNA) samples were not contaminated with genomic DNA. The primers used are listed in Table 1.

### 4.9. CatWalk Automated Gait Analysis

The CatWalk automated gait assay (Noldus, Wageningen, NL, Netherlands) was described elsewhere [69,70]. The behavioral response of a leg muscle injury can be related to the surface pressure exerted by the injured leg, which is reflected in the paw–floor contact area and paw pressure intensity. Briefly, mice traversed a walkway (a 100 cm × 10 cm pathway) spontaneously in a darkened room. Light was projected onto the long margin of the glass floor of the walkway and internally reflected within the floor. When the paw of the animal touched the floor, the points of contact lit up, and a wide-angle camera placed under the walkway recorded the light intensity generated by the footprint. The camera detected the average light intensity to within a pixel of the total paw-floor contact area. The signal intensity depended on the degree of paw–floor print area and increased as the applied pressure increased. More pressure exerted resulted in a larger paw–floor contact area, which increased the paw pressure pixel intensities and paw-floor print area. The intensities were digitized using a PClmage-SG frame grabber board (Matrix Vision GmbH, Oppenheimer, Germany). The Noldus CatWalk XT8.1 software system acquired, compressed, stored, and analyzed the videotapes as the mice crossed the walkway. The mice used in this study had no prior experience walking the pathway and did not hesitate to cross it. Gait parameters from the right and left sides of either front or hind paws were used for subsequent data analysis. For each animal, the paw pressure intensity or the walking paw–floor print area was expressed as the mean ± SD or transformed into the percentage of the value obtained from the contralateral uninjured limb (defined as 100%) of each animal.

### 4.10. Statistical Analyses

Data were expressed as means ± SD and were analyzed using the one-way ANOVA module of the Prism 4.02 software (GraphPad Software, San Diego, CA, USA). Tukey’s tests were used to assess whether the differences between the experimental results for paired groups were significant, and Dunnett’s test was applied to individually compare the results for three groups after the significance was found using the F-test. The Student’s *t*-test was used when two samples were compared.

## 5. Conclusions 

Muscle injury presents a challenging traumatological problem because injured muscles heal slowly and often do not completely recover. Currently, the best therapy available is simply supportive care. The muscle repair process depends on a balance between muscle regeneration and fibrosis, which is influenced by the inflammatory response. This unprecedented discovery provides innovative evidence for dissecting the mechanisms of uMSCs in healing muscle injury. The uMSCs displayed an innovative role in ameliorating early-onset neutrophil infiltration and activation, and in attenuating neutrophil-mediated chemotaxis to facilitate the attenuation of acute inflammation. This helped to protect against extensive muscle damage and subsequent muscle fibrosis, endorsing subsequent functional recovery. The administration of a stem-cell-based strategy by employing of uMSCs may effectively impact several innovative pathways involved in the pathogenesis of skeletal muscle injury.

## Figures and Tables

**Table 1:** Primer list *.

Gene	NCBI Ref. No	Primer	Sequence
			
Human *Myf5*	NM_005593	Forward	GCTGCCAGTTCTCACCTTCT
		Reverse	CACGTGCTCGTCCTCATCT
Human *MyoD*	NM_002478	Forward	TCTCTGCTCCTTTGCCACAAC
		Reverse	GAGTGCTCTTCGGGTTTCAG
Human *Myogenin*	NM_002479	Forward	AGGTGTGTAAGAGGAAGTCGG
		Reverse	AGGCGCTCGATGTACTGGA
Human *18S ribosomal RNA (rRNA)*	NR_003286	Forward	GTGTGCCTACCCTACG
		Reverse	TGACCCGCACTTACTC
Mouse *GAPDH*	NM_008084	Forward	TTGTGATGGGTGTGAACCAC
		Reverse	GTCATGAGCCCTTCCACAAT
Mouse *Fn1*	NM_001276413.1	Forward	AGGAAGCCGAGGTTTTAACTG
		Reverse	AGGACGCTCATAAGTGTCACC
Mouse *COL1A1*	NM_009912	Forward	ATGGATTCCCGTTCGAGTACG
		Reverse	TCAGCTGGATAGCGACATCG
Mouse *COL10A1*	NM_009925	Forward	GCCAAGCAGTCATGCCTGAT
		Reverse	GACACGGGCATACCTGTTACC
Mouse *TGF-β*	NM_011577	Forward	GAAGGACCTGGGTTGGAAGT
		Reverse	TGGTTGTAGAGGGCAAGGAC

* All primers were designed for both conventional and quantitative PCR.
